# Enteric Pathogens in children under five with diarrhoea in a rotavirus vaccinated population in Coastal Ghana, 2023

**DOI:** 10.1371/journal.pone.0356904

**Published:** 2026-08-28

**Authors:** Delia Akosua Bandoh, Mawuli Dzodzomenyo, Ernest Kenu, Francis Ekow Dennis, Duah Dwomoh, Edwin Andrew Afari, George Armah

**Affiliations:** 1 Department of Epidemiology and Disease Control, University of Ghana School of Public Health, Legon, Accra, Ghana; 2 Department of Biological, Environmental and Occupational Health Sciences, University of Ghana School of Public Health, Legon, Accra, Ghana; 3 Department of Electron Microscopy and Histopathology, Noguchi Memorial institute for Medical Research, University of Ghana, Accra, Ghana; 4 Department of Biostatistics, University of Ghana School of Public Health, Legon, Accra, Ghana; Università degli Studi di Parma: Universita degli Studi di Parma, ITALY

## Abstract

**Background:**

Despite the introduction and wide coverage of vaccination regimes such as rotavirus vaccination globally, diarrhoea remains as a major public health problem with new studies showing an emerging pattern in its etiology. We assessed the etiology of diarrhoea in a population of children under five years appropriately vaccinated against rotavirus.

**Methods:**

We conducted a cross-sectional study of children under five years reporting with diarrhoea at the district health facility in the Anloga district, Ghana. Stool samples of diarrhoea cases were tested for pathogens and co-infections using standard ELISA methods and TAQMAN Array Card RT-PCR Technology. The Vesikari Clinical Severity Scoring System was used to classify diarrhoea severity. Data generated was described descriptively.

**Results:**

Median age was 21.5 (IQR:12,30)months, 55.3%(21/38) with being male. About half of the cases were moderate diarrhoea severity (20/38). The Rotavirus A prevalence was 2.5%. The pathogen with the highest prevalence was *Enteroaggregative Escherichia coli (*EAEC) (52.6%, 20/38), *Shigella/ Enteroinvasive Escherichia coli* (Shigella/EIEC (76.3%, 29/38), Norovirus (26.3%, 10/38) and Giardia was the main parasite identified. The rate of co-infection was high (94.7%, 36/38). Co-infections with bacteria-virus present had more moderate-severe diarrhoea cases (73.3%, 11/15). Norovirus was present in most of the severe cases (75%, 3/4).

**Conclusion:**

The leading diarrhoea pathogens found were EAEC, Shigella/EIEC and Norovirus. The rate of pathogen co-infection was very high with bacteria-virus related co-infections affecting diarrhoea severity. The study recommends periodic diarrhoea pathogen surveillance through Ghana Health Service collaborating with research institutions such as Noguchi Memorial Institute for Medical Research (NMIMR). Also, enhancing water, sanitation and hygiene practices at the district, community and individual levels as a means of diarrhoea prevention in the absence of additional vaccines for other diarrhoea pathogens.

## Introduction

Over the years, diarrhoea has remained a disease of public health importance with vulnerable groups such as children under five years bearing the greatest brunt, recording more than 1.7 billion cases each year [1, 2]. The known transmission pathway of diarrhoea has been pathogens transported to host through contaminated food or water and person to person fecal-oral transmissions. Developing countries like Ghana with water and sanitation problems therefore record the highest number of cases [3,4]. Therefore, the main interventions used in such settings to reduce diarrhoea incidence have been improving water, sanitation and hygiene (WASH) and vaccination against pathogens.

One of the main virulent pathogens, rotavirus has been known to be the major cause of diarrhoea morbidity and mortality among children under five globally [5–8]. Thus, the introduction of the rotavirus vaccine 20 years ago, led to a decline in diarrhoea cases [9]. This has also been confirmed by different studies on aetiology of diarrhoea in under-fives in sub-Saharan Africa which reported a decline in rotavirus cases and attributed it to vaccine effectiveness and changes in the circulating rotavirus strain [10–12]. It is widely known that the presence of multiple enteropathogens can affect the severity of gastrointestinal illnesses [13,14]. However, in recent years, new patterns in the aetiology of diarrhoea are emerging. Some studies on diarrhoea in children under five years in India and China observed changes in diarrhoea pathogens and concluded on that the changing pattern of diarrhoea [15,16]

Since Ghana adopted the rotavirus A vaccine in 2012 as part of the routine immunizations for infants, coverage has been very high with a 94% coverage in 2022, yet diarrhoea in this age group still persists with a prevalence of 13% in 2022 [17]. A baseline study conducted in the Anloga district of the volta region record a 30% prevalence of reported diarrhoea in the past 2 weeks among children under five [18]. In this same setting, over 75% of the children had been fully vaccinated with over 80% having access to improved water and sanitation. These findings and other similar ones from other settings in Ghana emphasize the need for intensifying the surveillance of circulating diarrhoea pathogens to ensure effective management, and also identifying location-specific etiological agents to reduce the diarrhoea burden among children under five years. We therefore assessed diarrhoea severity and pathogens present in children under five with diarrhoea in a rotavirus vaccinated population, in a coastal district in Ghana. This study provides data on the aetiologies of diarrhoea, which in most parts of the country is not available for clinical decision making.

## Methods

### Study design

We conducted a cross-sectional study of under-fives reporting with diarrhoea at the district health facility in the Anloga district, Ghana from November 2022 to August 2023. This laboratory assessment identified diarrhoea pathogen in the stool of children presenting with diarrhoea. Stool samples of diarrhoea cases were collected and tested using standard ELISA methods and TAQMAN Array Card RT-PCR Technology.

### Setting

The Anloga district is a coastal district in the Volta region of Ghana. The district has a population of 94,895 [19] and is located east of the Volta estuary, with about 60% of the total land area covered by Lagoon and the Volta River, [20]. The district has six health centers, four functional CHPS compounds and three private facilities serving their health needs.

The sources of drinking water in the district are river, well, standpipes, dugout and borehole. However, the major sources of domestic water supply to the people in the district is pipe borne water. More than a quarter of households (29.1%) utilise public toilets (water closets or KVIP).

### Study population and case definitions

The case definition used for diarrhoea in this study were adapted from the IDSR 3^rd^ edition.

*Suspected case: Passage of 3 or more loose or watery stools in the past 24 hours with or without dehydration and: Some dehydration -- two or more of the following signs: restlessness, irritability; sunken eyes; thirst; skin pinch goes back slowly, or Severe dehydration -- two or more of the following signs: lethargy or unconsciousness; sunken eyes; not able to drink or drinking poorly; skin pinch goes back very slowly* [21].

Using this definition, any child under five years attending a health facility in Anloga district and confirmed to have met the case definition for diarrhoea was eligible for the study. Confirmation of the presence of diarrhoea was done by the health worker in charge of the facility. Cases were identified at the health facility. Caregivers who consented to provide stool samples were enrolled into the study. Enrollment and sample collection was done over an 8-month period.

### Sample size calculation

Using the prevalence of rotavirus in Ghana [22], and the number of cases to be recruited into the study, Prevalence = 12% cases = 171, a minimum sample of 83 was obtained. The total sample size of 83 was obtained and tested for rotavirus A pathogen. Further molecular analysis was conducted with 40 randomly sampled cases to determine their pathogen diversity.

### Data collection

At the health facility, children under five years who reported with diarrhoea were referred by the Physician Assistants and nurses on duty to the study team. The study was then explained to caregivers. Those who agreed signed a consent form and were enrolled into the study. Information on child age and sex, child immunization status of the child, child schooling, WASH practices, were also collected through structured interviews with the caregivers. After the interview, caregivers were asked to provide stool samples from their children using sterile containers and return containers to the interviewer.

### Lab sample collection and transportation

Collected stool samples were well labelled with interviewee codes, stored below 2 Celsius and transported to the laboratory for pathogen detection.

### Lab procedures

All 80 samples were processed and tested for rotavirus antigen using the ProSpecT Rotavirus Microplate Assay for Detection of Rotavirus Antigen in Stool Specimens for the E Enzyme-Linked Immunosorbent assay (ELISA). Samples were processed and tested for rotavirus antigen using the ProSpecT Rotavirus Microplate Assay for Detection of Rotavirus Antigen in Stool Specimens for the E Enzyme-linked immunoassay (ELISA). For the test, a 10% stool suspension was prepared according to the manufacturer’s instructions. The aliquots were prepared using phosphate buffered saline. A peanut size of stool weighing roughly 0.1 mg was used to prepare 1 ml aliquot. For stools that were liquid, 100µl of stool was used in the preparation. The procedure for testing was followed as prescribed by the manufacturer’s instructions [23]. The ProSpect test kit was used because of its high sensitivity and specificity (sensitivity 99.2% and specificity 99.2) [23].

### TAQMAN Array Card (TAC) Laboratory Testing Procedure

After this, 38 of the 80 samples were randomly selected for molecular testing. We extracted total nucleic acid of the stool samples. This proportion was selected to identify any additional pathogens which may be present in the stool samples aside the known rotavirus. Pathogen detection was performed on 180 mg of each sample using quantitative polymerase chain reaction with customized TaqMan Array cards. The laboratory procedure was based on previously published works and manufacturer’s [24–26]. The tests were done with a customized Taqman Array card (Thermo Fisher, Carlsblad, CA, USA) which tests using probe-based qPCR assays of 23 enteropathogens. The total nucleic acid was extracted using the modified QIAmp Fast DNA Stool mini kit (Qiagen, Hilden, Germany). Prior to the extraction, a lysis buffer was prepared by Mixing InhibitEX Buffer thoroughly and bead beating with glass beads. Bacteriophage MS2for RNA and phocine herpesvirus for DNA extraction control PAHV and extraction control were added to each sample as external controls to track efficiency of nucleic-acid extraction and amplification. Lysing was followed by Protease treatment and purification through the spin column, and elution of the total nucleic acid. One well was left blank for the batch of tests to eliminate laboratory contamination. The TAQMAN Array Card (TAC) test was done following the manufacturer’s procedure [27]. The laboratory analysis was done following steps from Bandoh (2024)’s study protocol and Lappan [25,28].

### Data management and analysis

For the ELISA test, codes of samples that turned out positive were manually recorded in the Laboratory Microsoft Excel sheet first followed by negatives.

TAQMAN Array card results were analysed with QuantuStudio RealTime PCR software version 1.3. Cut off points were adjusted for target pathogens to correct thresholds. Data was then exported to Microsoft Excel 2016 and cleaned and by setting each standard cycle threshold (CT) for pathogens and genotypes. Pathogen level detection was set at 35 and genotype level detection set at 40. For pathogens, all CT values below 35 were set as positive, and above 35 set at negative, undetermined values were also set as negative. For genotypes, all CT values below 40 were set were set as positive, and above 40 set at negative, undetermined values were also set as negative. CT positives and negatives were converted to binary outcomes (0 = negative and 1 = positive). Frequencies of pathogen types classified as bacteria, virus and parasites, pathogen co-infections and distribution by age and sex pathogens were generated with Stata Version 17. Results were presented in tables and figures.

### Diarrhoea severity classification

Using the Vesikari Clinical Severity Scoring System, diarrhoea cases were classified as mild, moderate or severe. Parameters used for the classification were diarrhoea duration and frequency, vomiting, level of dehydration and treatment given [29] (Tables 1 and 2). Severity is classified based on generated scores out of a total of 20 across the different parameter categories.

**Table 1:** Parameter and scores for Vesikari Clinical Severity Scoring System.

	Score		
Parameter	1	2	3
Diarrhoea			
Maximum number stools per day(frequency)	1-3	4-5	6
Diarrhoea duration (days)	1-4	5	6
Vomiting duration (day)	1	2	5
Temperature (in degrees Celcius)	37.1 -38.4	38.5-38.9	39.0>
Dehydration (%)	N/A	1-5%	>5%
Treatment	Rehydration	Hospitalisation	N/A
**Rating Scale for Vesikari Clinical Severity Scoring System Severity (Maximum score 20)**			
**Severity category**	**Score**		
Mild	<7		
Moderate	7 -10		
Severe	11		

Adopted from Lewis 2011 [29]

**Table 2:** Demographics of caregivers and children by diarrhoea severity.

Variable	Mild N(%)	Moderate N(%)	Severe N(%)
	14(36.8)	20 (52.6)	4(10.5)
Age of child (months)			
<1years	2 (14.3)	6 (30.0)	1 (25.0)
1year	5 (35.7)	5 (25.0)	2 (50.0)
2years	3 (21.4)	5 (25.0)	1 (25.0)
3years	1 (7.1)	3 (15.0)	0 (0.0)
4years	3 (21.4)	1 (5.0)	0 (0.0)
Sex of child			
Male	8 (57.1)	9 (45.0)	3 (75.0)
Female	6 (42.9)	11 (55.0)	1 (25.0)
Level of education of caregiver			
No formal education	2 (14.3)	1 (5.0)	2 (50.0)
Primary	5 (35.7)	3 (15.0)	1 (25.0)
Middle/JHS	5 (35.7)	10 (50.0)	1 (25.0)
SHS/commercial	2 (14.3)	5 (25.0)	0 (0.0)
Tertiary	0 (0.0)	1 (5.0)	0 (0.0)
Age of caregiver (in complete years)			
<20years	0 (0.0)	1 (5.0)	0 (0.0)
20-30years	6 (42.9)	12 (60.0)	3 (75.0)
31-40years	6 (42.9)	6 (30.0)	1 (25.0)
41-49years	2 (14.3)	1 (5.0)	0 (0.0)
Primary occupation of caregiver			
Unemployed	3 (21.4)	3 (15.0)	3 (75.0)
Formally employed	1 (7.1)	1 (5.0)	0 (0.0)
Fisherfolks	1 (7.1)	0 (0.0)	0 (0.0)
Trader	3 (21.4)	8 (40.0)	1 (25.0)
Artisan	6 (42.9)	8 (40.0)	0 (0.0)
Wealth			
Poor	6 (42.9)	6 (30.0)	1 (25.0)
Middle	4 (28.6)	8 (40.0)	1 (25.0)
Rich	4 (28.6)	6 (30.0)	2 (50.0)

The mean for the ages of children in months was calculated and frequencies generated for categorical variables such as type of diarrhoea and pathogen type. Diarrhoea cases were described by severity, pathogen type, vaccination status, access to water and sanitation services. Chi-square and Fischer’s exact tests were used to test for association between these categorical characteristics and diarrhoea severity at 95% confidence interval. The analysis compared each intervention to the severity of diarrhoea recorded. Additionally, diarrhoea severity was described descriptively by the various types of pathogens present. These were presented in text, tables and charts.

### Ethical considerations

The proposal for this study was granted approval by the Ghana Health Service Ethics Review Committee (GHS-ERC 020/07/22). Permission was obtained from the regional and district health directorates and the District Assembly. Permission was obtained from the community leaders and health facility in-charges. The purpose of the study was explained to all participants in detail and their questions answered before enrolment. Participants were assured of confidentiality. Informed consent forms were administered to all study participants prior to participation. Participants signed or thumb printed the informed consent documents before any interview was conducted. Participation in all sections of the study was voluntary and subjects were informed that they can withdraw at any time they wish even after consent had been given and during participating in the study. All information obtained from the study was kept confidential on password protected computers.

## Results

### Description of children

Median age was 21.5 (IQR:30,12) months, 55.3%(21/38) with being male. About half of the cases (20/38) were of moderate severity. More males (75%:3/4) had severe diarrhoea compared to females (Table 2).

### Description of WASH & vaccination status

Almost all, children (97.4%; 37/38) had full rotavirus vaccination and had access to improved water sources. Majority of caregivers reported practicing handwashing but most of them had moderate diarrhoea (52%, 14/23) and most children 89%(30/38) did not use household toilet facility. Majority of the sever cases (75%, ¾) did not use household toilets. Differences were not found to be significant (Table 3).

**Table 3:** Diarrhoea Severity by Access to WASH Interventions.

Variable	Mild N(%)	Moderate N(%)	Severe N(%)
N	14(36.8)	20 (52.6)	4(10.5)
Main source of drinking water			
Unimproved	2 (14.3)	0 (0.0)	0 (0.0)
Improved	12 (85.7)	20 (100.0)	4 (100.0)
Type of household toilet facility			
Unimproved	3 (21.4)	7 (35.0)	0 (0.0)
Improved	11 (78.6)	13 (65.0)	4 (100.0)
Handwashing practice			
Good	9 (64.3)	14 (70.0)	4 (100)
Poor	5(35.7)	6 (30.0)	0 (0)
Child use of household toilet			
No	19 (71.4)	16 (80.0)	3 (75)
Yes	4 (28.6)	4 (20.0)	1 (25.0)
Rotavirus vaccination			
No	0 (0.0)	1 (5.0)	0 (0.0)
Yes	14 (100.0)	19 (95.0)	4 (100.0)

### Pathogen description

**Fig 1:**
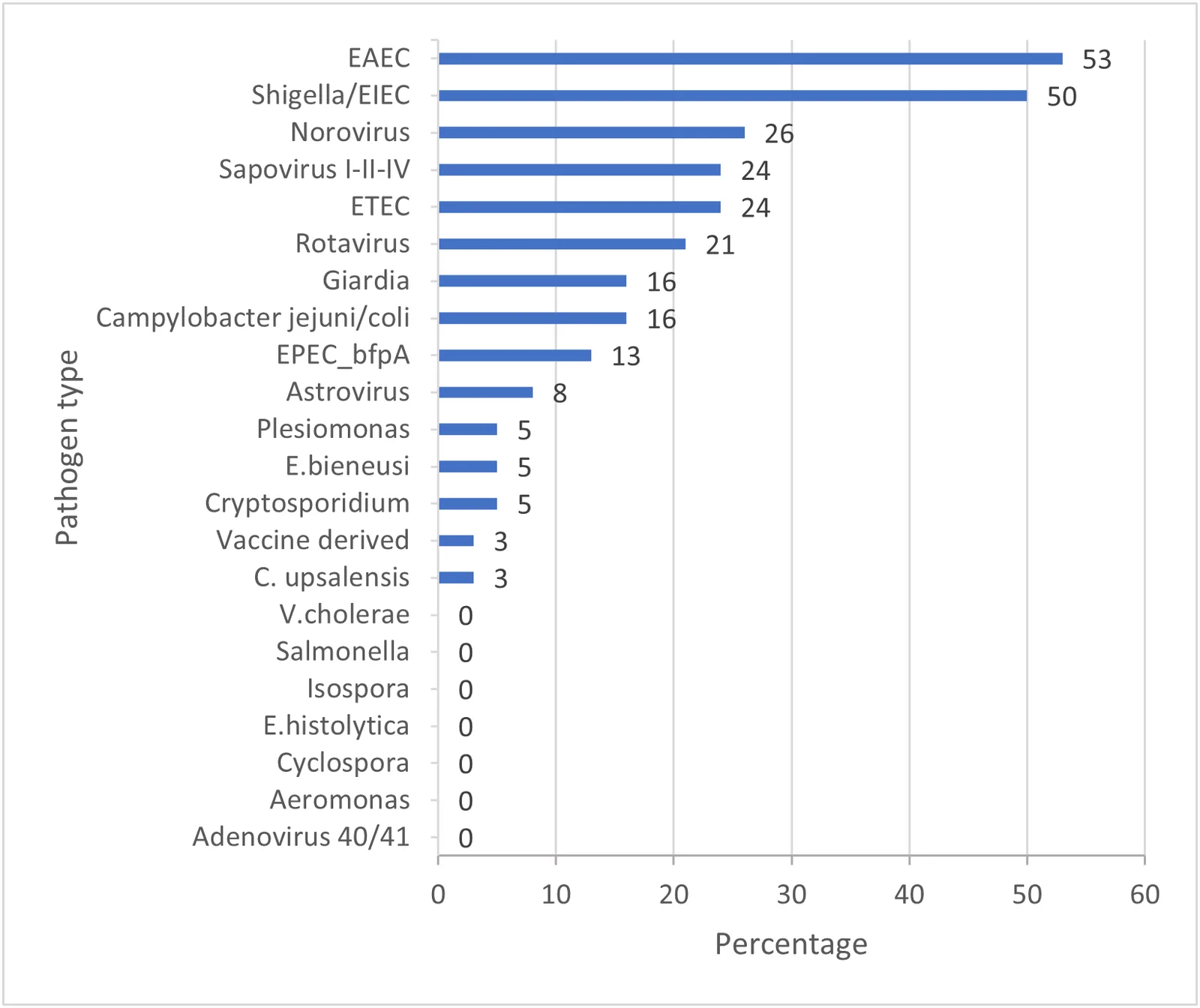
Pathogen testing results for samples using TaqMan Array Card.

Diarrhoea pathogens were found in 94.7% (36/38) of the samples. Other viruses identified were Norovirus(26.3%, 10/38); Saprovirus (23.7%, 9/38); bacteria pathogens identified included: Shigella/ Enteroinvasive *Escherichia coli* (50%, 19/38); Enterotoxigenic *Escherichia coli*(23.7%, 9/38); Enteroaggregative *Escherichia coli*(52.6%, 20/38) whiles Giardia (16%, 6/38) was the main parasite identified. The pathogen with the highest prevalence was *Enteroaggregative Escherichia coli (*EAEC) (53%), *Shigella/ Enteroinvasive Escherichia coli* (Shigella/EIEC (50%) and Norovirus (26%) (Fig 1).

For bacteria, *Campylobacter upsalensis* was found only in children under two years. *Campylobacter jejuni/coli* was present children of both age groups with children under two having the highest proportion (18.18%, 4/22). For parasites, giardia was the most common parasite found in children of all ages with most being above 2 years (18.8%, 3/16) and least common among under twos (13.6%, 3/22). Cyclospora, *Entamoeba histolytica* and Isospora (Cystoisospora) were not found among any of the age groups. For virus, astrovirus was found in only children above two years 18.8% (3/16), while norovirus was found in more children under two years (36.4%, 8/22) than those above two years (12.5%, 2/16). The Rotarix vaccine strain was found in one child above two years (1/16). Rotavirus was found in all the age groups. There were no significant differences seen in all pathogens by age groups (p > 0.05). (Table 4).

**Table 4:** Frequency of Bacteria pathogens tested using TAQMAN Array Card by age in children under five years, Anloga District, 2023.

N	2 years and below (%)	Above 2 years (%)	p-value*
	22	16	
	n (%)	n (%)	
**Bacteria**			
Aeromonas	0 (0.00)	0 (0.00)	
*Campylobacter upsalensis*	1 (4.55)	0 (0.00)	1.00
*Campylobacter jejuni/coli*	4 (18.18)	2 (12.50)	1.00
*Enteroaggregative Escherichia coli*	12 (54.55)	8 (50.00)	1.00
*Enteropathogenic Escherichia coli* (bfpA)	3 (13.64)	2 (12.50)	1.00
*Enterotoxigenic Escherichia coli*	6 (27.27)	3 (18.75)	0.71
Plesiomonas	1 (4.55)	1 (6.25)	1.00
Salmonella	0 (0.00)	0 (0.00)	
*Shigella/ Enteroinvasive Escherichia coli*	8 (36.36)	11 (68.75)	0.099
*Vibrio cholerae*	0 (0.00)	0 (0.00)	
**Parasite**			
Cryptosporidium	1 (4.55)	1 (6.25)	1.00
Cyclospora	0 (0.00)	0 (0.00)	
*Enterocytozoon bieneusi*	1 (4.55)	1 (6.25)	1.00
*Entamoeba histolytica*	0 (0.00)	0 (0.00)	
Giardia	3 (13.64)	3 (18.75)	0.68
Isospora (Cystoisospora)	0 (0.00)	0 (0.00)	
**Virus**			
Adenovirus 40/41	0 (0.00)	0 (0.00)	
Astrovirus	0 (0.00)	3 (18.75)	0.066
Norovirus	8 (36.36)	2 (12.50)	0.14
Rotarix vaccine	0 (0.00)	1 (6.25)	0.42
RotaTeq vaccine	0 (0.00)	0 (0.00)	
Rotavirus	2 (9.09)	6 (37.50)	0.050
Sapovirus I-II-IV	6 (27.27)	5 (31.25)	1.00

*p-values obtained from Fischer’s Exact test

Half of the cases with four or more co-infections (50%, 7/14) had mild diarrhoea. Co-infections with bacteria-virus present had more moderate-severe diarrhoea cases (73.3%, 11/15). An equal proportion of cases with one-two pathogens (15.4, 2/13) and cases with four or more pathogens present (15.4, 2/13) experienced severe diarrhoea respectively. (Table 5).

**Table 5:** Diarrhoea severity and pathogen co-infection.

Variable	Severity of Diarrhoea (n = 38)		
	Mild N(%)	Moderate N(%)	Severe N(%)
N	**14(36.8)**	**20 (52.6)**	**4(10.5)**
One or two	4 (30.8)	7 (53.9)	2 (15.4)
Three	3 (33.3)	6 (66.7)	0 (0)
Four more	7(50.0)	5 (35.7)	2 (14.9)
Pathogen groupings*			
Bacteria	4 (50)	3 (37.5)	1 (12.5)
Bacteria-parasite	2 (100)	0 (0)	0 (0)
Virus	0(0)	2 (66.7)	1 (33.3)
Virus-parasite	1 (100)	0 (0)	0 (0)
Virus-bacteria	4 (26.7)	10 (66.7)	1 (6.7)
Virus-bacteria-parasite	3 (36.8)	3 (36.8)	1 (14.3)

*p-values obtained from Fischer’s Exact test

### Description of severe diarrhoea cases

Most severe cases (75%,3/4) were in their second year of life (above 12 months). Norovirus was in almost all (3/4) severe diarrhoea cases. Campylobacter was found in half (2/4) of the severe cases. More males had severe diarrhoea than females (Table 6)

**Table 6:** Pathogens found in severe diarrhoea samples.

Case	Age (months)	Sex	Type of pathogens	Pathogens
1	14	Female	Bacteria, virus, parasite	• Campylobacter • EPEC • Giardia • Norovirus
2	11	Male	Virus	• Norovirus
3	17	Male	Bacteria, virus, parasite	• Campylobacter • Cupsalensis • Norovirus
4	25	Male	Bacteria	• EACE • Shigella/EIEC

## Discussion

We assessed diarrhoea severity and pathogens present in children under five with diarrhoea in a rotavirus vaccinated population in a coastal district in Ghana. Most of the diarrhoea cases in the study presented with moderate case diarrhoea cases with the most common pathogens of public health importance being *Enteroaggressive Escherichia Coli* (EAEC) and *Escherichia Coli/Shigella* and Norovirus. These pathogens identified according to WHO form part of the major causes of diarrhoea morbidity and mortality among children under five years globally [30]. Though recognized as pathogens of public health importance, rotavirus A vaccine remains the only available vaccine in Ghana and other settings. Additionally, in Ghana, the diarrhoea pathogen surveillance gives more attention to bacteria pathogen than viruses [31]. Thus, viruses like norovirus and saprovirus are not extensively monitored. Therefore, high proportion of these pathogens in this population suggests the need to revisit preventive intervention to serve as a barrier to prevent children from being infected by these pathogens

As expected for a highly vaccinated population, there was a very low proportion of cases with rotavirus A identified. This is a sign that rotavirus vaccination the children in the population received was effective. This finding confirms several studies in children under five which found that rotavirus vaccination reduced the incidence of diarrhoea in children in this age group [5,6,32–34].

The rate of co-infection among the tested samples was high with approximately eight in ten tested samples having multiple enteric pathogen combinations. Co-infection with two or more enteric pathogens during early childhood is known to be a common phenomenon especially in developing countries and have been reported in several studies in Cameroun, South Africa, and other LMICs [13,35–38]. These diarrhoea pathogens co-infection in developing countries has generally been attributed to poor hygiene and sanitation conditions within these environments [35,39]. Diarrhoeal pathogen co‑infections pose a significant challenge in children and may lead to increased severity of the illness. This is likely due to the synergistic interactions among the multiple pathogens present. [36]. Cases with bacteria-viral co-infections generally had more moderate diarrhoea severity. Additionally, Norovirus was common among severe diarrhoea cases. Studies have found Norovirus to be associated with high burden of diarrhoea and a significant cause of diarrhoea gastroenteritis, (a type of diarrhoea disease) in African children [35,40].

In the light of the diarrhoea severity, understanding the etiological role of each pathogen becomes essential since it impacts treatment effectiveness of diarrhoea [35]. This points out the need to focus on the current diarrhoea pathogens being identified in the setting. Though this study did not assess explicitly asymptotic carriage of diarrheal pathogen among the study subjects, studies in Guinea Bissau and Papua New Guinea found a high proportion of healthy populations with pathogens without any symptoms suggestive of diarrhoea [41,42]. Thus, the high rate of co-infections observed in this study may not entirely reflect simultaneous active infection by multiple pathogens but rather, may represent one clinically active pathogen superimposed on an existing asymptomatic carrier state for one or more organisms. Future studies incorporating matched asymptomatic controls to separate true co-infection from background carriage in this setting are therefore encouraged. This would provide a more accurate picture of the etiological burden of each pathogen identified.

The observed pathogens seem to show a changing trend in the known aetiology of diarrhoea among children under five years. Diarrhoeagenic *Escherichia Coli* strains and norovirus prevalence seems to be on the increase compared to than that of rotavirus. Therefore, relying on solely rotavirus vaccination with little consideration for effectively tailored WASH interventions which break transmission in the first place as a means of

dealing with the emerging diarrhoea pathogens is essential. Though the rotavirus vaccine intervention has contributed significantly to solving the diarrhoea problem, there still remains work to be done to further reduce the diarrhoea burden in children under five years. There is the need to focus on vaccines for other diarrhoea pathogens as they increase.

Some limitations to this study include small sample size of the study making it difficult to make inference. The short duration of the study making it unable to collect samples for more than 12 calendar months. Additionally, the TAC assays done alone were unable to fully tell if all pathogens present accounted for the diarrhoea or each pathogen’s contribution. However, the study covered the major seasons of the year and was an exploratory study providing an overview of diarrhoea pathogens in children under five years in the setting. Thus, without making inferences, it provides a picture of the pathogens present in the setting, addressing the goal of the paper.

Summarizing the study, we observed high rate of diarrhoea pathogen co-infections with most of the cases reporting with moderate diarrhoea points to the need to reconsider the existing interventions in place to break the faecel-oral transmission pathway of diarrhoea among children under five years. Given the absence of other diarrhoea vaccines, interim measures such as WASH interventions to prevent the pathogens from being ingested by the children such as improved water, hygiene and sanitation practices centered on children under five years need to be intensified. Additionally, long-term recommendations could consider diarrhoea vaccines which target other pathogens in addition to Rotavirus A.

## Conclusion

In conclusion, among the studied population, the leading pathogen for diarrhoea was not rotavirus A but rather Diarrhoeagenic EAEC, Shigella/EIEC and norovirus. Additionally, the rate of co-infection was high with bacteria related co-infections having diarrhoea severity. The study recommends that Ghana Health Service to engage in periodic diarrhoea pathogen surveillance and creation of sentinel sites throughout the country through collaborations with research institutions such as Noguchi Memorial Institute for Medical Research (NMIMR) to provide insight on the circulating pathogens. Also, in the absence of additional vaccines for other diarrhoea pathogens, the district assembly and stakeholders in charge of WASH should focus on implementing strategies which aid the breaking of diarrhoea pathogen transmission such as more frequent household inspections with focus on water, sanitation and hygiene practices at the community and individual specifically in diarrhoea control.
