# Supplementary material for: Enteric Pathogens in children under five with diarrhoea in a rotavirus vaccinated population in Coastal Ghana, 2023
Source: PLoS One. 2026 Aug 28;21(8):e0356904. doi: 10.1371/journal.pone.0356904 (PMC13524243; doi:10.1371/journal.pone.0356904)
Supplement: Supplementary file 2 [file pone.0356904.s002.docx]

**Reviewer report for enteric pathogens in children under five with diarrhoea in a vaccinated population in coastal Ghana, 2023**

Dear Editor

Thank you very much for your comments. The review has improved our manuscript

Kindly find attached a point by point response of the comments raised by booth the Editor and reviewers.

Regards

Corresponding Author

Journal Requirements:

When submitting your revision, we need you to address these additional requirements.

1.Please ensure that your manuscript meets PLOS ONE's style requirements, including those for file naming. The PLOS ONE style templates can be found at

<https://journals.plos.org/plosone/s/file?id=wjVg/PLOSOne_formatting_sample_main_body.pdf> and

<https://journals.plos.org/plosone/s/file?id=ba62/PLOSOne_formatting_sample_title_authors_affiliations.pdf><<https://track.editorialmanager.com/CL0/https:%2F%2Fjournals.plos.org%2Fplosone%2Fs%2Ffile%3Fid=ba62%2FPLOSOne_formatting_sample_title_authors_affiliations.pdf/1/010f019dfaf9eb43-b13eb342-466d-4c83-a824-6e85050647f1-000000/1XJI0h43ePvM9U08Hx-JTDMiz0Y6J1g2iaB1Qh695hk=258>>
 **Response**

Thank you for the comment, the manuscript has been reformatted to the journal’s specification

2. We are unable to open your Supporting Information file “STROBE-checklist-v4-combined-Plos pathogens.docx” Please kindly revise as necessary and re-upload.

**Response**

The STROBE checklist has been reloaded

3. Please include captions for your Supporting Information files at the end of your manuscript, and update any in-text citations to match accordingly. Please see our Supporting Information guidelines for more information: <http://journals.plos.org/plosone/s/supporting-information><<http://track.editorialmanager.com/CL0/http:%2F%2Fjournals.plos.org%2Fplosone%2Fs%2Fsupporting-information/1/010f019dfaf9eb43-b13eb342-466d-4c83-a824-6e85050647f1-000000/swlc8fxhbAcJv0HFQjUIerldrWiRV3-MuJkDPKKkAlc=258>>.

**Response**

The authors have no supporting information files. The files uploaded were the human participant research checklist and the STROBE checklist

4. If the reviewer comments include a recommendation to cite specific previously published works, please review and evaluate these publications to determine whether they are relevant and should be cited. There is no requirement to cite these works unless the editor has indicated otherwise.

**Response**

Thank you for the comment. Authors would consider before accepting the citation of papers suggested by the reviewers.

Additional Editor Comments:

The submitted article presents interesting aspects, however it suffers from the need for improvement in the description of the methodological approach and the clarity of the text, in accordance with the reviewers' observations, which must be promptly addressed.

**Response**

Thank you Editor, reviewer comments have been fully addressed to improve the quality of the paper.

Reviewer's Responses to Questions

Comments to the Author

1. Is the manuscript technically sound, and do the data support the conclusions?

The manuscript must describe a technically sound piece of scientific research with data that supports the conclusions. Experiments must have been conducted rigorously, with appropriate controls, replication, and sample sizes. The conclusions must be drawn appropriately based on the data presented.

Reviewer #1: Yes

Reviewer #2: Partly

________________________________

2. Has the statistical analysis been performed appropriately and rigorously?

Reviewer #1: Yes

Reviewer #2: I Don't Know

________________________________

3. Have the authors made all data underlying the findings in their manuscript fully available?

The PLOS Data policy<<http://track.editorialmanager.com/CL0/http:%2F%2Fwww.plosone.org%2Fstatic%2Fpolicies.action%23sharing/1/010f019dfaf9eb43-b13eb342-466d-4c83-a824-6e85050647f1-000000/jeGdZO_o9H7s4wf35nDYii3olP6F8TjqxdivG4Udu9Q=258>> requires authors to make all data underlying the findings described in their manuscript fully available without restriction, with rare exception (please refer to the Data Availability Statement in the manuscript PDF file). The data should be provided as part of the manuscript or its supporting information, or deposited to a public repository. For example, in addition to summary statistics, the data points behind means, medians and variance measures should be available. If there are restrictions on publicly sharing data—e.g. participant privacy or use of data from a third party—those must be specified.

Reviewer #1: No

Reviewer #2: Yes

________________________________

4. Is the manuscript presented in an intelligible fashion and written in standard English?

PLOS ONE does not copyedit accepted manuscripts, so the language in submitted articles must be clear, correct, and unambiguous. Any typographical or grammatical errors should be corrected at revision, so please note any specific errors here.

Reviewer #1: No

Reviewer #2: No

________________________________

5. Review Comments to the Author

Please use the space provided to explain your answers to the questions above. You may also include additional comments for the author, including concerns about dual publication, research ethics, or publication ethics. (Please upload your review as an attachment if it exceeds 20,000 characters)

Reviewer #1: GENERAL: It is good to see this study on diarrheal disease pathogens from a Ghanian team. The results are interesting despite being limited in terms of where samples were obtained from the small size of the study. The presentation of the data is reasonable, but there are some confusing things in the study. It needs a good proofread for grammatical and typographical errors. Use of scientific nomenclature for organism names is very inconsistent throughout. I have not highlighted the typographical and grammatical errors except in a few places.

The main limitation of the discussion is that it doesn't consider asymptomatic carriage of diarrheal disease pathogens. This is never discussed as part of the reason for co-infections or carriage of different pathogens.

Overall, the study is of interest but needs quite a lot of work to get it into shape for publication.

**Response**

Thank you for the comment, the discussion has been modified, the entire paper has been proofread and grammatical errors addressed. Methods section and data presentation have been improved. The discussion has been revised to capture the asymptomatic carriage of diarrhoea pathogens

SPECIFIC:
Line 29: 'Under fives' is not a noun - please correct.

**Response**

This has been corrected

L36: 'The rotavirus prevalence was...'

**Response**

“the” has been added to the statement

L55: Person to person transmission of diarrheal disease pathogens important too.
**Response**

This has been added

L105-111: It is confusing to have two case definitions that are slightly different. How did you use them? It looks like the IDSR suspected case definition is more restrictive than the WHO one.
**Response**

Thank you for pointing this out. Cases were recruited at the health facility using the IDSR case definition since that is stand practice for all health facilities in Ghana. This case definition has therefore been maintained

L115: Here controls are mentioned, which is confusing as they are never mentioned again and results are not presented elsewhere. Was this a case control study?
response

**Response**

No, this was not a case-control study. The use of the term “Control samples” referred to laboratory procedure. This has been written out clearly to avoid confusion.

L137: Note that 38/50 isn't 50%, it is 47.5%. I haven't checked all calculations, but it is concerning to see these simple mistakes in a manuscript.

**Response**

This has been corrected and the actual numbers rather stated to prevent overestimation. All proportions have also been recalculated and presented appropriately. Thank you

L193: Do you know how many cases of diarrhea occurred in the health facilities during the study period, ie - what proportion of children under 5 presenting with diarrhea were enrolled in your study?

Response

No, the study did not collect information on the number of diarrhoea cases reporting to the health facility. Rather, cases of diarrhoea in children under five years were referred to the data collectors stationed at the health facilities during the study period. Given that is was a study, caregivers were at liberty to take part. Some opted out after enrollment because they were required to provide stool samples.

This forms part of a bigger study on evaluating WASH interventions and the risk factors of diarrhoea. the study enrolled 193 cases in total over a 14 month period, however, some caregivers opted out of the part of the aetiological study due to varied reasons

L205: It is unclear why such a high proportion of children did not use household toilet if the majority of households had improved toilet facilities.
**Response**

The setting is a peri urban setting where most households share toilet facilities with multiple houses. Generally, Children are therefore not introduced to these facilities at an early stage till they are above three years.

L287: I think it is a bit strong to say that the pathogens you identified represent a 'paradigm shift'.
**response**

This has been replaced with a “changing trend”

L303: Please include a more detailed section on limitations of the study. At the moment there is only two sentences, which don't really address proper limitations. In my mind, the limitations are that you can't really tell from PCR tests what pathogens are causing diarrhea. Another significant limitation is that we don't really have information on how study subjects got into the study.

**response**

Thank you, limitations have been revised to include limitations of the PCR test, the duration of the study and the sample size.

The recruitment section has been revised to indicate how study subjects were enrolled.

Reviewer #2: Dear author , the data has a very good information for the added value on enteropathogen scientific community though the way you presented them and your discussions and conclusion needs some review for this paper to be published . Kindly find the attached comments

________________________________

6. PLOS authors have the option to publish the peer review history of their article (what does this mean?<<https://track.editorialmanager.com/CL0/https:%2F%2Fjournals.plos.org%2Fplosone%2Fs%2Feditorial-and-peer-review-process%23loc-peer-review-history/1/010f019dfaf9eb43-b13eb342-466d-4c83-a824-6e85050647f1-000000/K0Rcd4dcLvwZLGagIdfuL5MstTthTQOFsdxWGQCcvk0=258>>). If published, this will include your full peer review and any attached files.

If you choose “no”, your identity will remain anonymous but your review may still be made public.

Do you want your identity to be public for this peer review? For information about this choice, including consent withdrawal, please see our Privacy Policy<<https://track.editorialmanager.com/CL0/https:%2F%2Fwww.plos.org%2Fprivacy-policy/1/010f019dfaf9eb43-b13eb342-466d-4c83-a824-6e85050647f1-000000/lneQsvg6wn4mApAlIKYL3yEfb0pTEUArRWjqFVgJY7M=258>>.

Reviewer #1: No

Reviewer #2: No

________________________________

**Dear Author**

This manuscript presents a cross-sectional study investigating the etiological agents of diarrhoea among rotavirus-vaccinated children under five in coastal Ghana. The study addresses an important public health question regarding shifting diarrhoeal pathogen patterns in the post-rotavirus vaccine era. The use of molecular diagnostics (TaqMan Array Card) strengthens the scientific value.

However, there are **substantial issues related to clarity, methodological consistency, data presentation, and language quality** that must be addressed before the manuscript can be considered for publication.

**Reviewer Comment**

**Title**: It highlights the vaccinated population without being specific. I would suggest saying “Enteric Pathogens in children under five with diarrhoea in a **rotavirus** vaccinated population in Coastal Ghana, 2023

**For study population and case definition: There is a big confusion in the study designs , it stated it’s a cross-sectional but here there is the sentence which explains cases and controls, can you put it in a way someone can understand who was the control and this study involded children under five and yet you said you consented caregivers -did they produce stools, was the stools analysed and presented? Even in the results section I looked at the control results but was not found can you clarify was it case control study or cross-sectional?- Cases were identified at the health facility. Whiles controls were recruited from the vicinity of the cases. Children under five years who had lived in the district for less than 6 months were excluded because they were considered to have different levels of exposure. Caregivers who consented to provide stool samples were enrolled into the study. Enrollment and sample collection was done over an 8-month period.**

**Response**

The title has been modified as proposed by the reviewer to: Enteric Pathogens in children under five with diarrhoea in a rotavirus vaccinated population in Coastal Ghana, 2023.

This has been rewritten for clarity: Even though the reviewer comment mentioned a case control study design, the manuscript did not state anywhere the design was a case-control study. The design was a cross sectional study where stool samples were collected from children who reported with diarrhoea at the health facility for further testing, after consent was obtained from caregivers. This component was part of a bigger study and is reported as such.. The case definition stated defined the population of study subjects from which samples were taken from.

Reviewer’s Comment

From line 122-124 you stated sample size and sampling, it is not very clear how the 40 samples for TAC was selected, clarify please.

**Response**

The samples for the TAC were selected random from the sample population. Specifically, the full list of samples were entered into Microsoft Excel. Using the random number generation function, 40 numbers were selected for the TAC array test. Two samples had to be further dropped because in running the TAC each batch of samples has to be run with a blank well and another well with only the master mix to serve as internal controls for the entire batch of tests being run. The two samples were dropped using the same Microsoft Excel Random number generation process.

Reviewer’s Comment

For the laboratory procedure, could you explain how the 38 samples selected like did you take all the rotavirus positive elisas plus others or how did you judge whome to include inTAC testing.

**Response**

No the positives were not added purposively, in selection of samples for the TAC, all samples sent to the laboratory (including the 2 which tested positive for rotavirus A) were added in the Microsoft Excel random number generation command.

Reviewer’s Comment

As this was more of molecular testing could you write down briefly how the Nucleic acid was extracted, manufacture for the kit then for the Realtime PCR you can explain briefly on how TAC was set which kits were used and what what were you targeting- I mean pathogens of interest .

**Response**

Laboratory procedure was revised with the text below: Methods section, pages 7-8

After this, 38 of the 80 samples were randomly selected for molecular testing. We extracted total nucleic acid of the stool samples. This proportion was selected to identify any additional pathogens which may be present in the stool samples aside the known rotavirus. Pathogen detection was performed on 180mg of each sample using quantitative polymerase chain reaction with customized TaqMan Array cards. The laboratory procedure was based on previously published works and manufacturer’s [25], [26], [27]. The tests were done with a customized Taqman Array card (Thermo Fisher, Carlsblad, CA, USA) which tests using probe-based qPCR assays of 23 enteropathogens. The total nuliec acid was extracted using the modified QIAmp Fast DNA Stool mini kit (Qiagen, Hilden, Germany). Prior to the extraction, a lysis buffer was prepared by Mixing InhibitEX Buffer thoroughly and bead beating with glass beads. Bacteriophage MS2for RNA and phocine herpesvirus for DNA extraction control PAHV and extraction control were added to each sample as external controls to track efficiency of nucleic-acid extraction and amplification. Lysing was followed by Protease treatment and purification through the spin column, and elution of the total nucleic acid. One well was left blank for the batch of tests to eliminate laboratory contamination. The TAQMAN Array Card (TAC) test was done following the manufacturer’s procedure [28]. The laboratory analysis was done following steps from Bandoh (2024)’s study protocol and Lappan [26], [29].

Reviewer’s Comment

From line 137-151 needs to be written scientifically, TAC is a PCR and Quantistudio is a Machine for running this TAC assay and not for data analysis. After the Tac results are put in an Excel, it is when you deal with data management and analysis I would wish if you read a paper from JIE LIU 2016 on enteric pathogen detection, at least to adapt the writing from nucleic acid extraction to Taqman array card real-time PCR process until results interpretation**.**

**Response**

Thank you for the suggestion, the paper has been a very helpful resource

Data management and alanyls has been revised as follows:

Cycle threshold information generated by QuantuStudio RealTime PCR software version 1.3 was exported to Microsoft Excel 2016. Cut off points were adjusted for target pathogens to correct thresholds. This was done by setting each standard cycle threshold (CT) for pathogens and genotypes. Pathogen level detection was set at 35 and genotype level detection set at 40. For pathogens, all CT values below 35 were set as positive, and above 35 set at negative, undetermined values were also set as negative. For genotypes, all CT values below 40 were set as positive, and above 40 set at negative, undetermined values were also set as negative. CT positives and negatives were converted to binary outcomes (0= negative and 1= positive). Frequencies of pathogen types classified as bacteria, virus and parasites, pathogen co-infections and distribution by age and sex pathogens were generated with Stata Version 17. Results were presented in tables and figures.

**Reviewer’s CommentsFor the results**

Table 5 needs to be more precise: the title is written ….. and pathogen co-infection. I expected to see data for co-infection only, but it was combined with single-pathogen detection.

**Response**

Table has been revised. This currently shows only co-infections

Correct line 242 tittle with the explanations 243-245 and the table 5 citation is not true.

**Response**

The title has been corrected and reference made to table 6 instead of table 5

Table 6 is very confusing I suggest it to be more organized

**Response**

Table 6 has been reorganized for clarity

From line 234-237 cited table 4 which do not have those data talked about severity.

**Response**

The typo has been corrected. The narration refers rather to table 5 which presents ‘Diarrhoea severity and pathogen co-infection’

Have a good table that summarises well the pathogens that co-infect the one you have are not clear.

**Response**

Table 5 has been reanalyzed and currently reports only cases with co-infections ( 2 or more pathogens). Thank you

**Reviewer Comment**

**DISCUSSION**

The severity discussion is not critical for the discussion as the mild and moderate diarrhoea are more alarming than the one with only 2 individuals which is same as one-two.

In your co-infection table it idid not show the bacteria-bacteria combination, which you gave credit and said it has more severity than virus and parasite.

**Response**

Thank you for the observation.

The discussion has also been revised based on comments provided. Based on reanalysis and review of the data, that statement has been corrected. The table in the results has also been revised
