# Supplementary material for: Enteric Pathogens in children under five with diarrhoea in a rotavirus vaccinated population in Coastal Ghana, 2023
Source: PLoS One. 2026 Aug 28;21(8):e0356904. doi: 10.1371/journal.pone.0356904 (PMC13524243; doi:10.1371/journal.pone.0356904)
Supplement: Supplementary file 3 [file pone.0356904.s001.docx]

REVIEWER REPORT FOR Enteric Pathogens in children under five with diarrhoea in a vaccinated population in Coastal Ghana, 2023

**Dear Author**

This manuscript presents a cross-sectional study investigating the etiological agents of diarrhoea among rotavirus-vaccinated children under five in coastal Ghana. The study addresses an important public health question regarding shifting diarrhoeal pathogen patterns in the post-rotavirus vaccine era. The use of molecular diagnostics (TaqMan Array Card) strengthens the scientific value.

However, there are **substantial issues related to clarity, methodological consistency, data presentation, and language quality** that must be addressed before the manuscript can be considered for publication.

**Title**: It highlights the vaccinated population without being specific. I would suggest saying “Enteric Pathogens in children under five with diarrhoea in a **rotavirus** vaccinated population in Coastal Ghana, 2023

**For study population and case definition: There is a big confusion in the study designs , it stated it’s a cross-sectional but here there is the sentence which explains cases and controls, can you put it in a way someone can understand who was the control and this study involded children under five and yet you said you consented caregivers -did they produce stools, was the stools analysed and presented? Even in the results section I looked at the control results but was not found can you clarify was it case control study or cross-sectional?- Cases were identified at the health facility. Whiles controls were recruited from the vicinity of the cases. Children under five years who had lived in the district for less than 6 months were excluded because they were considered to have different levels of exposure. Caregivers who consented to provide stool samples were enrolled into the study. Enrollment and sample collection was done over an 8-month period.**

From line 122-124 you stated sample size and sampling, it is not very clear how the 40 samples for TAC was selected, clarify please.

For the laboratory procedure, could you explain how are the 38 samples selected like did you take all the rotavirus positive elisas plus others or how did you judge whome to include inTAC testing.

As this was more of molecular testing could you write down briefly how the Nucleic acid was extracted, manufacture for the kit then for the Realtime PCR you can explain briefly on how TAC was set which kits were used and what what were you targeting- I mean pathogens of interest .

From line 137-151 needs to be written scientifically, TAC is a PCR and Quantistudio is a Machine for running this TAC assay and not for data analysis. After the Tac results are put in an Excel, it is when you deal with data management and analysis I would wish if you read a paper from JIE LIU 2016 on enteric pathogen detection, at least to adapt the writing from nucleic acid extraction to Taqman array card real-time PCR process until results interpretation**.**

**For the results**

Table 5 needs to be more precise: the title is written ….. and pathogen co-infection. I expected to see data for co-infection only, but it was combined with single-pathogen detection.

Correct line 242 tittle with the explanations 243-245 and the table 5 citation is not true.

Table 6 is very confusing I suggest it to be more organized

From line 234-237 cited table 4 which do not have those data talked about severity.

Have a good table that summarises well the pathogens that co-infect the one you have are not clear.

**DISCUSSION**

The severity discussion is not critical for the discussion as the mild and moderate diarrhoea are more alarming than the one with only 2 individuals which is same as one-two.

In your co-infection table it idid not show the bacteria-bacteria combination, which you gave credit and said it has more severity than virus and parasite.
