# Supplementary material for: Enteric Pathogens in children under five with diarrhoea in a rotavirus vaccinated population in Coastal Ghana, 2023
Source: PLoS One. 2026 Aug 28;21(8):e0356904. doi: 10.1371/journal.pone.0356904 (PMC13524243; doi:10.1371/journal.pone.0356904)
Supplement: Peer review history

# Author comment: Author response to Decision Letter 0

**DOI:** 10.1371/journal.pone.0356904.r001

**Transfer Alert**

This paper was transferred from another journal. As a result, its full editorial history (including decision letters, peer reviews and author responses) may not be present.

21 Jan 2026

# Peer review: Decision Letter 0

**DOI:** 10.1371/journal.pone.0356904.r002

**Authors:** Flora De Conto

5 May 2026

Dear Dr.  Dzodzomenyo,

Thank you for submitting your manuscript to PLOS ONE. After careful consideration, we feel that it has merit but does not fully meet PLOS ONE’s publication criteria as it currently stands. Therefore, we invite you to submit a revised version of the manuscript that addresses the points raised during the review process.

Please submit your revised manuscript by Jun 19 2026 11:59PM. If you will need more time than this to complete your revisions, please reply to this message or contact the journal office at plosone@plos.org. When you're ready to submit your revision, log on to https://www.editorialmanager.com/pone/ and select the 'Submissions Needing Revision' folder to locate your manuscript file.

- A letter that responds to each point raised by the academic editor and reviewer(s). You should upload this letter as a separate file labeled 'Response to Reviewers'.
- A marked-up copy of your manuscript that highlights changes made to the original version. You should upload this as a separate file labeled 'Revised Manuscript with Track Changes'.
- An unmarked version of your revised paper without tracked changes. You should upload this as a separate file labeled 'Manuscript'.

If you would like to make changes to your financial disclosure, please include your updated statement in your cover letter. Guidelines for resubmitting your figure files are available below the reviewer comments at the end of this letter.

If applicable, we recommend that you deposit your laboratory protocols in protocols.io to enhance the reproducibility of your results. Protocols.io assigns your protocol its own identifier (DOI) so that it can be cited independently in the future. For instructions see: https://journals.plos.org/plosone/s/submission-guidelines#loc-laboratory-protocols. Additionally, PLOS ONE offers an option for publishing peer-reviewed Lab Protocol articles, which describe protocols hosted on protocols.io. Read more information on sharing protocols at https://plos.org/protocols?utm_medium=editorial-email&utm_source=authorletters&utm_campaign=protocols.

As the corresponding author, your ORCID iD is verified in the submission system and will appear in the published article. PLOS supports the use of ORCID, and we encourage all coauthors to register for an ORCID iD and use it as well. Please encourage your coauthors to verify their ORCID iD within the submission system before final acceptance, as unverified ORCID iDs will not appear in the published article. *Only*  the individual author can complete the verification step; PLOS staff *cannot*  verify ORCID iDs on behalf of authors.

We look forward to receiving your revised manuscript.

Kind regards,

Flora De Conto, Ph.D.

Academic Editor

PLOS One

Journal Requirements:

When submitting your revision, we need you to address these additional requirements.

1.Please ensure that your manuscript meets PLOS ONE's style requirements, including those for file naming. The PLOS ONE style templates can be found at

https://journals.plos.org/plosone/s/file?id=wjVg/PLOSOne_formatting_sample_main_body.pdf and

https://journals.plos.org/plosone/s/file?id=ba62/PLOSOne_formatting_sample_title_authors_affiliations.pdf

2. We are unable to open your Supporting Information file “STROBE-checklist-v4-combined-Plos pathogens.docx” Please kindly revise as necessary and re-upload.

3. Please include captions for your Supporting Information files at the end of your manuscript, and update any in-text citations to match accordingly. Please see our Supporting Information guidelines for more information: http://journals.plos.org/plosone/s/supporting-information.

4. If the reviewer comments include a recommendation to cite specific previously published works, please review and evaluate these publications to determine whether they are relevant and should be cited. There is no requirement to cite these works unless the editor has indicated otherwise.

Additional Editor Comments:

The submitted article presents interesting aspects, however it suffers from the need for improvement in the description of the methodological approach and the clarity of the text, in accordance with the reviewers' observations, which must be promptly addressed.

[Note: HTML markup is below. Please do not edit.]

Reviewer's Responses to Questions

**Comments to the Author**

1. Is the manuscript technically sound, and do the data support the conclusions?

Reviewer #1: Yes

Reviewer #2: Partly

**********

2. Has the statistical analysis been performed appropriately and rigorously?

Reviewer #1: Yes

Reviewer #2: I Don't Know

**********

3. Have the authors made all data underlying the findings in their manuscript fully available?

The PLOS Data policy

Reviewer #1: No

Reviewer #2: Yes

**********

4. Is the manuscript presented in an intelligible fashion and written in standard English?

Reviewer #1: No

Reviewer #2: No

**********

Reviewer #1: GENERAL: It is good to see this study on diarrheal disease pathogens from a Ghanian team. The results are interesting despite being limited in terms of where samples were obtained from the small size of the study. The presentation of the data is reasonable, but there are some confusing things in the study. It needs a good proofread for grammatical and typographical errors. Use of scientific nomenclature for organism names is very inconsistent throughout. I have not highlighted the typographical and grammatical errors except in a few places. The main limitation of the discussion is that it doesn't consider asymptomatic carriage of diarrheal disease pathogens. This is never discussed as part of the reason for co-infections or carriage of different pathogens. Overall, the study is of interest but needs quite a lot of work to get it into shape for publication.

SPECIFIC:

Line 29: 'Under fives' is not a noun - please correct.

L36: 'The rotavirus prevalence was...'

L55: Person to person transmission of diarrheal disease pathogens important too.

L105-111: It is confusing to have two case definitions that are slightly different. How did you use them? It looks like the IDSR suspected case definition is more restrictive than the WHO one.

L115: Here controls are mentioned, which is confusing as they are never mentioned again and results are not presented elsewhere. Was this a case control study?

L137: Note that 38/50 isn't 50%, it is 47.5%. I haven't checked all calculations, but it is concerning to see these simple mistakes in a manuscript.

L193: Do you know how many cases of diarrhea occurred in the health facilities during the study period, ie - what proportion of children under 5 presenting with diarrhea were enrolled in your study?

L205: It is unclear why such a high proportion of children did not use household toilet if the majority of households had improved toilet facilities.

L287: I think it is a bit strong to say that the pathogens you identified represent a 'paradigm shift'.

L303: Please include a more detailed section on limitations of the study. At the moment there is only two sentences, which don't really address proper limitations. In my mind, the limitations are that you can't really tell from PCR tests what pathogens are causing diarrhea. Another significant limitation is that we don't really have information on how study subjects got into the study.

Reviewer #2: Dear author , the data has a very good information for the added value on enteropathogen scientific community though the way you presented them and your discussions and conclusion needs some review for this paper to be published . Kindly find the attached comments

**********

what does this mean?). If published, this will include your full peer review and any attached files.

If you choose “no”, your identity will remain anonymous but your review may still be made public.

**Do you want your identity to be public for this peer review?**  For information about this choice, including consent withdrawal, please see our Privacy Policy

Reviewer #1: No

Reviewer #2: No

**********

[NOTE: If reviewer comments were submitted as an attachment file, they will be attached to this email and accessible via the submission site. Please log into your account, locate the manuscript record, and check for the action link "View Attachments". If this link does not appear, there are no attachment files.]

To ensure your figures meet our technical requirements, please review our figure guidelines: https://journals.plos.org/plosone/s/figures

You may also use PLOS’s free figure tool, NAAS, to help you prepare publication quality figures: https://journals.plos.org/plosone/s/figures#loc-tools-for-figure-preparation.

NAAS will assess whether your figures meet our technical requirements by comparing each figure against our figure specifications.

Attachment Submitted filename: REPORT FOR Enteric Pathogens in children Ghana.docx

# Author comment: Author response to Decision Letter 1

**DOI:** 10.1371/journal.pone.0356904.r003

30 Jun 2026

Dear Editor

Thank you very much for your comments. The review has improved our manuscript

Kindly find attached a point by point response of the comments raised by booth the Editor and reviewers.

Regards

Corresponding Author

Journal Requirements:

When submitting your revision, we need you to address these additional requirements.

1.Please ensure that your manuscript meets PLOS ONE's style requirements, including those for file naming. The PLOS ONE style templates can be found at

https://journals.plos.org/plosone/s/file?id=wjVg/PLOSOne_formatting_sample_main_body.pdf and

https://journals.plos.org/plosone/s/file?id=ba62/PLOSOne_formatting_sample_title_authors_affiliations.pdf<https://track.editorialmanager.com/CL0/https:%2F%2Fjournals.plos.org%2Fplosone%2Fs%2Ffile%3Fid=ba62%2FPLOSOne_formatting_sample_title_authors_affiliations.pdf/1/010f019dfaf9eb43-b13eb342-466d-4c83-a824-6e85050647f1-000000/1XJI0h43ePvM9U08Hx-JTDMiz0Y6J1g2iaB1Qh695hk=258>

Response

Thank you for the comment, the manuscript has been reformatted to the journal’s specification

2. We are unable to open your Supporting Information file “STROBE-checklist-v4-combined-Plos pathogens.docx” Please kindly revise as necessary and re-upload.

Response

The STROBE checklist has been reloaded

3. Please include captions for your Supporting Information files at the end of your manuscript, and update any in-text citations to match accordingly. Please see our Supporting Information guidelines for more information: http://journals.plos.org/plosone/s/supporting-information<http://track.editorialmanager.com/CL0/http:%2F%2Fjournals.plos.org%2Fplosone%2Fs%2Fsupporting-information/1/010f019dfaf9eb43-b13eb342-466d-4c83-a824-6e85050647f1-000000/swlc8fxhbAcJv0HFQjUIerldrWiRV3-MuJkDPKKkAlc=258>.

Response

The authors have no supporting information files. The files uploaded were the human participant research checklist and the STROBE checklist

4. If the reviewer comments include a recommendation to cite specific previously published works, please review and evaluate these publications to determine whether they are relevant and should be cited. There is no requirement to cite these works unless the editor has indicated otherwise.

Response

Thank you for the comment. Authors would consider before accepting the citation of papers suggested by the reviewers.

Additional Editor Comments:

The submitted article presents interesting aspects, however it suffers from the need for improvement in the description of the methodological approach and the clarity of the text, in accordance with the reviewers' observations, which must be promptly addressed.

Response

Thank you Editor, reviewer comments have been fully addressed to improve the quality of the paper.

Reviewer's Responses to Questions

Comments to the Author

1. Is the manuscript technically sound, and do the data support the conclusions?

The manuscript must describe a technically sound piece of scientific research with data that supports the conclusions. Experiments must have been conducted rigorously, with appropriate controls, replication, and sample sizes. The conclusions must be drawn appropriately based on the data presented.

Reviewer #1: Yes

Reviewer #2: Partly

________________________________

2. Has the statistical analysis been performed appropriately and rigorously?

Reviewer #1: Yes

Reviewer #2: I Don't Know

________________________________

3. Have the authors made all data underlying the findings in their manuscript fully available?

The PLOS Data policy<http://track.editorialmanager.com/CL0/http:%2F%2Fwww.plosone.org%2Fstatic%2Fpolicies.action%23sharing/1/010f019dfaf9eb43-b13eb342-466d-4c83-a824-6e85050647f1-000000/jeGdZO_o9H7s4wf35nDYii3olP6F8TjqxdivG4Udu9Q=258> requires authors to make all data underlying the findings described in their manuscript fully available without restriction, with rare exception (please refer to the Data Availability Statement in the manuscript PDF file). The data should be provided as part of the manuscript or its supporting information, or deposited to a public repository. For example, in addition to summary statistics, the data points behind means, medians and variance measures should be available. If there are restrictions on publicly sharing data—e.g. participant privacy or use of data from a third party—those must be specified.

Reviewer #1: No

Reviewer #2: Yes

________________________________

4. Is the manuscript presented in an intelligible fashion and written in standard English?

PLOS ONE does not copyedit accepted manuscripts, so the language in submitted articles must be clear, correct, and unambiguous. Any typographical or grammatical errors should be corrected at revision, so please note any specific errors here.

Reviewer #1: No

Reviewer #2: No

________________________________

5. Review Comments to the Author

Please use the space provided to explain your answers to the questions above. You may also include additional comments for the author, including concerns about dual publication, research ethics, or publication ethics. (Please upload your review as an attachment if it exceeds 20,000 characters)

Reviewer #1: GENERAL: It is good to see this study on diarrheal disease pathogens from a Ghanian team. The results are interesting despite being limited in terms of where samples were obtained from the small size of the study. The presentation of the data is reasonable, but there are some confusing things in the study. It needs a good proofread for grammatical and typographical errors. Use of scientific nomenclature for organism names is very inconsistent throughout. I have not highlighted the typographical and grammatical errors except in a few places.

The main limitation of the discussion is that it doesn't consider asymptomatic carriage of diarrheal disease pathogens. This is never discussed as part of the reason for co-infections or carriage of different pathogens.

Overall, the study is of interest but needs quite a lot of work to get it into shape for publication.

Response

Thank you for the comment, the discussion has been modified, the entire paper has been proofread and grammatical errors addressed. Methods section and data presentation have been improved. The discussion has been revised to capture the asymptomatic carriage of diarrhoea pathogens

SPECIFIC:

Line 29: 'Under fives' is not a noun - please correct.

Response

This has been corrected

L36: 'The rotavirus prevalence was...'

Response

“the” has been added to the statement

L55: Person to person transmission of diarrheal disease pathogens important too.

Response

This has been added

L105-111: It is confusing to have two case definitions that are slightly different. How did you use them? It looks like the IDSR suspected case definition is more restrictive than the WHO one.

Response

Thank you for pointing this out. Cases were recruited at the health facility using the IDSR case definition since that is stand practice for all health facilities in Ghana. This case definition has therefore been maintained

L115: Here controls are mentioned, which is confusing as they are never mentioned again and results are not presented elsewhere. Was this a case control study?

response

Response

No, this was not a case-control study. The use of the term “Control samples” referred to laboratory procedure. This has been written out clearly to avoid confusion.

L137: Note that 38/50 isn't 50%, it is 47.5%. I haven't checked all calculations, but it is concerning to see these simple mistakes in a manuscript.

Response

This has been corrected and the actual numbers rather stated to prevent overestimation. All proportions have also been recalculated and presented appropriately. Thank you

L193: Do you know how many cases of diarrhea occurred in the health facilities during the study period, ie - what proportion of children under 5 presenting with diarrhea were enrolled in your study?

Response

No, the study did not collect information on the number of diarrhoea cases reporting to the health facility. Rather, cases of diarrhoea in children under five years were referred to the data collectors stationed at the health facilities during the study period. Given that is was a study, caregivers were at liberty to take part. Some opted out after enrollment because they were required to provide stool samples.

This forms part of a bigger study on evaluating WASH interventions and the risk factors of diarrhoea. the study enrolled 193 cases in total over a 14 month period, however, some caregivers opted out of the part of the aetiological study due to varied reasons

L205: It is unclear why such a high proportion of children did not use household toilet if the majority of households had improved toilet facilities.

Response

The setting is a peri urban setting where most households share toilet facilities with multiple houses. Generally, Children are therefore not introduced to these facilities at an early stage till they are above three years.

L287: I think it is a bit strong to say that the pathogens you identified represent a 'paradigm shift'.

response

This has been replaced with a “changing trend”

L303: Please include a more detailed section on limitations of the study. At the moment there is only two sentences, which don't really address proper limitations. In my mind, the limitations are that you can't really tell from PCR tests what pathogens are causing diarrhea. Another significant limitation is that we don't really have information on how study subjects got into the study.

response

Thank you, limitations have been revised to include limitations of the PCR test, the duration of the study and the sample size.

The recruitment section has been revised to indicate how study subjects were enrolled.

Reviewer #2: Dear author , the data has a very good information for the added value on enteropathogen scientific community though the way you presented them and your discussions and conclusion needs some review for this paper to be published . Kindly find the attached comments

________________________________

6. PLOS authors have the option to publish the peer review history of their article (what does this mean?<https://track.editorialmanager.com/CL0/https:%2F%2Fjournals.plos.org%2Fplosone%2Fs%2Feditorial-and-peer-review-process%23loc-peer-review-history/1/010f019dfaf9eb43-b13eb342-466d-4c83-a824-6e85050647f1-000000/K0Rcd4dcLvwZLGagIdfuL5MstTthTQOFsdxWGQCcvk0=258>). If published, this will include your full peer review and any attached files.

If you choose “no”, your identity will remain anonymous but your review may still be made public.

Do you want your identity to be public for this peer review? For information about this choice, including consent withdrawal, please see our Privacy Policy<https://track.editorialmanager.com/CL0/https:%2F%2Fwww.plos.org%2Fprivacy-policy/1/010f019dfaf9eb43-b13eb342-466d-4c83-a824-6e85050647f1-000000/lneQsvg6wn4mApAlIKYL3yEfb0pTEUArRWjqFVgJY7M=258>.

Reviewer #1: No

Reviewer #2: No

________________________________

Dear Author

This manuscript presents a cross-sectional study investigating the etiological agents of diarrhoea among rotavirus-vaccinated children under five in coastal Ghana. The study addresses an important public health question regarding shifting diarrhoeal pathogen patterns in the post-rotavirus vaccine era. The use of molecular diagnostics (TaqMan Array Card) strengthens the scientific value.

However, there are substantial issues related to clarity, methodological consistency, data presentation, and language quality that must be addressed before the manuscript can be considered for publication.

Reviewer Comment

Title: It highlights the vaccinated population without being specific. I would suggest saying “Enteric Pathogens in children under five with diarrhoea in a rotavirus vaccinated population in Coastal Ghana, 2023

For study population and case definition: There is a big confusion in the study designs , it stated it’s a cross-sectional but here there is the sentence which explains cases and controls, can you put it in a way someone can understand who was the control and this study involded children under five and yet you said you consented caregivers -did they produce stools, was the stools analysed and presented? Even in the results section I looked at the control results but was not found can you clarify was it case control study or cross-sectional?- Cases were identified at the health facility. Whiles controls were recruited from the vicinity of the cases. Children under five years who had lived in the district for less than 6 months were excluded because they were considered to have different levels of exposure. Caregivers who consented to provide stool samples were enrolled into the study. Enrollment and sample collection was done over an 8-month period.

Response

The title has been modified as proposed by the reviewer to: Enteric Pathogens in children under five with diarrhoea in a rotavirus vaccinated population in Coastal Ghana, 2023.

This has been rewritten for clarity: Even though the reviewer comment mentioned a case control study design, the manuscript did not state anywhere the design was a case-control study. The design was a cross sectional study where stool samples were collected from children who reported with diarrhoea at the health facility for further testing, after consent was obtained from caregivers. This component was part of a bigger study and is reported as such.. The case definition stated defined the population of study subjects from which samples were taken from.

Reviewer’s Comment

From line 122-124 you stated sample size and sampling, it is not very clear how the 40 samples for TAC was selected, clarify please.

Response

The samples for the TAC were selected random from the sample population. Specifically, the full list of samples were entered into Microsoft Excel. Using the random number generation function, 40 numbers were selected for the TAC array test. Two samples had to be further dropped because in running the TAC each batch of samples has to be run with a blank well and another well with only the master mix to serve as internal controls for the entire batch of tests being run. The two samples were dropped using the same Microsoft Excel Random number generation process.

Reviewer’s Comment

For the laboratory procedure, could you explain how the 38 samples selected like did you take all the rotavirus positive elisas plus others or how did you judge whome to include inTAC testing.

Response

No the positives were not added purposively, in selection of samples for the TAC, all samples sent to the laboratory (including the 2 which tested positive for rotavirus A) were added in the Microsoft Excel random number generation command.

Reviewer’s Comment

As this was more of molecular testing could you write down briefly how the Nucleic acid was extracted, manufacture for the kit then for the Realtime PCR you can explain briefly on how TAC was set which kits were used and what what were you targeting- I mean pathogens of interest .

Response

Laboratory procedure was revised with the text below: Methods section, pages 7-8

After this, 38 of the 80 samples were randomly selected for molecular testing. We extracted total nucleic acid of the stool samples. This proportion was selected to identify any additional pathogens which may be present in the stool samples aside the known rotavirus. Pathogen detection was performed on 180mg of each sample using quantitative polymerase chain reaction with customized TaqMan Array cards. The laboratory procedure was based on previously published works and manufacturer’s [25], [26], [27]. The tests were done with a customized Taqman Array card (Thermo Fisher, Carlsblad, CA, USA) which tests using probe-based qPCR assays of 23 enteropathogens. The total nuliec acid was extracted using the modified QIAmp Fast DNA Stool mini kit (Qiagen, Hilden, Germany). Prior to the extraction, a lysis buffer was prepared by Mixing InhibitEX Buffer thoroughly and bead beating with glass beads. Bacteriophage MS2for RNA and phocine herpesvirus for DNA extraction control PAHV and extracti

Attachment Submitted filename: REPORT FOR Enteric Pathogens in children Ghana (1)_DB (1).docx

# Editor report: Decision Letter 1

**DOI:** 10.1371/journal.pone.0356904.r004

**Authors:** Flora De Conto

10 Aug 2026

Enteric Pathogens in children under five with diarrhoea in a rotavirus vaccinated population in Coastal Ghana, 2023

PONE-D-26-02594R1

Dear Dr. Dzodzomenyo,

We’re pleased to inform you that your manuscript has been judged scientifically suitable for publication and will be formally accepted for publication once it meets all outstanding technical requirements.

Within one week, you’ll receive an e-mail detailing the required amendments. When these have been addressed, you’ll receive a formal acceptance letter and your manuscript will be scheduled for publication.

An invoice will be generated when your article is formally accepted. Please note, if your institution has a publishing partnership with PLOS and your article meets the relevant criteria, all or part of your publication costs will be covered. Please make sure your user information is up-to-date by logging into Editorial Manager at Editorial Manager® and clicking the ‘Update My Information' link at the top of the page. For questions related to billing, please contact billing support.

If your institution or institutions have a press office, please notify them about your upcoming paper to help maximize its impact. If they’ll be preparing press materials, please inform our press team as soon as possible -- no later than 48 hours after receiving the formal acceptance. Your manuscript will remain under strict press embargo until 2 pm Eastern Time on the date of publication. For more information, please contact onepress@plos.org.

Kind regards,

Flora De Conto, Ph.D.

Academic Editor

PLOS One

Additional Editor Comments (optional):

Reviewers' comments:

# Editor report: Acceptance letter

**DOI:** 10.1371/journal.pone.0356904.r005

**Authors:** Flora De Conto

PONE-D-26-02594R1

PLOS One

Dear Dr. Dzodzomenyo,

I'm pleased to inform you that your manuscript has been deemed suitable for publication in PLOS One. Congratulations! Your manuscript is now being handed over to our production team.

At this stage, our production department will prepare your paper for publication. This includes ensuring the following:

* All references, tables, and figures are properly cited

* All relevant supporting information is included in the manuscript submission,

* There are no issues that prevent the paper from being properly typeset

You will receive further instructions from the production team, including instructions on how to review your proof when it is ready. Please keep in mind that we are working through a large volume of accepted articles, so please give us a few days to review your paper and let you know the next and final steps.

Lastly, if your institution or institutions have a press office, please let them know about your upcoming paper now to help maximize its impact. If they'll be preparing press materials, please inform our press team within the next 48 hours. Your manuscript will remain under strict press embargo until 2 pm Eastern Time on the date of publication. For more information, please contact onepress@plos.org.

You will receive an invoice from PLOS for your publication fee after your manuscript has reached the completed accept phase. If you receive an email requesting payment before acceptance or for any other service, this may be a phishing scheme. Learn how to identify phishing emails and protect your accounts at https://explore.plos.org/phishing.

If we can help with anything else, please email us at customercare@plos.org.

Thank you for submitting your work to PLOS One and supporting open access.

Kind regards,

PLOS ONE Editorial Office Staff

on behalf of

Prof. Flora De Conto

Academic Editor

PLOS One
